# Association Between Self-Reported Health and Reliance on Veterans Affairs for Health Care Among Veterans Affairs Enrollees

**DOI:** 10.1001/jamanetworkopen.2023.23884

**Published:** 2023-07-17

**Authors:** Liam Rose, Anna Schmidt, Elizabeth Gehlert, Laura A. Graham, Marion Aouad, Todd H. Wagner

**Affiliations:** 1 Health Economics Resource Center, VA Palo Alto Health Care System, Menlo Park, California; 2 Stanford Surgery Policy Improvement and Education Center, Stanford Medicine, Stanford, California; 3 Department of Economics, University of California, Irvine

## Abstract

This cross-sectional study using survey data investigates the association between level of reliance on the Department of Veterans Affairs for health care and self-reported health by type of insurance coverage among VA enrollees.

## Introduction

The Department of Veterans Affairs (VA) is the largest integrated health care system in the US. However, most enrollees (80%) have other health insurance.^1^ Veterans’ selective use of VA health care creates an immense challenge for VA leadership, the scale of which is relatively unknown. The VA allocates substantial resources to improve equitable access, prevent suicides, and provide transparency on hospital quality by analyzing VA-provided and VA-purchased care.^2,3^ These analyses will be inaccurate if patient health and reliance on the VA are linked, especially as the VA expands its role as a payer of care and the recently passed PACT Act brings in younger veterans, who are more likely to have private health insurance. This study investigated whether VA reliance was associated with self-reported health, a predictor of health outcomes,^4^ and whether this association varied for individuals with different types of outside insurance using a nationally representative survey.

## Methods

This cross-sectional study used data from the VA Survey of Enrollees from 2015 to 2021 (315 356 respondents). Race data were self-reported in the survey. This survey is fielded annually to a cross-section of approximately 50 000 VA-enrolled veterans. The survey asks about health insurance status, reliance on the VA for health care (“none,” “some,” “most,” or “all” health care), and self-reported health on a scale of 1 (very poor) to 5 (very good). We examined self-reported health by insurance status and self-reported VA reliance. We then used logistic regression to calculate the proportion of respondents reporting poor or very poor health by level of reliance on the VA within different insurance types. We weighted analyses using sampling weights designed to make the sample representative of enrollees in a given survey year (eAppendix in Supplement 1). This study was approved by the Stanford University institutional review board, and we followed the STROBE reporting guideline. We used R statistical software version 4.2.1 (R Project for Statistical Computing) and 2-sided *t* tests for statistical significance.

## Results

Survey respondents were representative of the VA population by design (91.5% male; mean [SD] age, 61 [17] years; 13.0% Black, 1.8% Asian, 78.9% White, and 6.2% other or unknown race). The Figure shows that insurance status was associated with large differences in self-reported health. VA enrollees with private coverage were the most likely (44.0%) and enrollees with Medicaid were the least likely (23.7%) to report being in good health. VA reliance was also associated with self-reported health. Veterans who received all care through the VA were the most likely to report poor health (33.3%), whereas 21.2% of veterans who received no VA care reported poor health. The adjusted analysis within insurance type (Table) showed similar findings. Apart from respondents with Medicaid and VA care only, individuals who reported no care with the VA were less likely to report poor health. Respondents with the VA and Medicare made up the largest group (124 674 respondents), among whom respondents with no VA reliance were 9.9 percentage points less likely to report poor health than those who used the VA for all health care needs.

**Figure.**
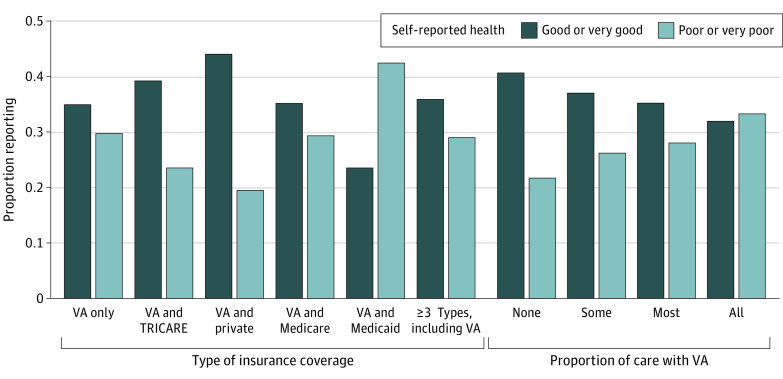
Self-Reported Health by Insurance Coverage and Proportion of Care Through the VA VA indicates US Department of Veterans Affairs.

**Table.** Estimated Proportion of VA Enrollees Reporting Poor or Very Poor Health by VA Reliance and Insurance Type

VA reliance level^a^	Proportion of enrollees, marginal difference (SE)^b^					
	With VA only	With VA and TRICARE	With VA and private insurance	With VA and Medicare	With VA and Medicaid	With ≥3 insurance types (including VA)
VA used for all needs, mean (SD)	0.321 (0.005)	0.322 (0.013)	0.243 (0.012)	0.352 (0.004)	0.407 (0.026)	0.369 (0.006)
Respondents, No.	31 575	4824	4102	42 758	1213	17 830
Most health care	−0.067 (0.011)	−0.067 (0.018)	−0.029 (0.016)	−0.055 (0.005)	0.070 (0.045)	−0.048 (0.009)
Respondents, No.	6984	3234	5267	27 896	690	17 659
Some health care	−0.029 (0.017)	−0.081 (0.018)	−0.025 (0.015)	−0.086 (0.005)	−0.034 (0.043)	−0.085 (0.008)
Respondents, No.	2629	3950	7480	38 458	479	32 894
None	−0.034 (0.023)	−0.158 (0.015)	−0.079 (0.014)	−0.099 (0.006)	0.088 (0.057)	−0.125 (0.008)
Respondents, No.	1017	3631	6548	15 562	239	18 889
Total respondents, No.	42 205	15 639	23 397	124 674	2621	87 272

Abbreviations: SE, standard error; VA, US Department of Veterans Affairs.

^a^ Reliance was measured by asking the survey respondent to finish the statement “I use VA to meet,” with the following options: all (ie, “all of my health care needs”), most (ie, “most of my health care needs”), some (ie, “some of my health care needs”), or none (ie, “none of my health care needs).”

^b^ SEs were calculated via the delta method. The first row shows the mean for the base group (respondents using the VA for all health care needs), which was used as the comparison group, and subsequent rows give marginal effects from a logistic regression for different levels of reliance on the VA. Regressions are weighted by survey weights.

## Discussion

This cross-sectional study using survey data found that VA enrollees who reported poor health relied more heavily on the VA for health care needs. This held true for individuals with various types of outside health insurance, apart from Medicaid recipients who were a small proportion of the VA population. This study highlights the difficulties of measuring population health among VA enrollees given that analyses using VA data will struggle to adequately address confounders when patients systematically select into VA care. This directly affects the VA’s stated goals of ensuring adequate access to care, preventing suicides, and supporting veterans’ whole health given that it remains unknown if resources allocated to improve access are targeting individuals in need or providing additional options to those in better health and with better outside insurance. The primary limitation of this study was the inability to establish drivers of VA reliance, which should be investigated in future research.

These results also highlight the difficulties of providing care, measuring quality, and forecasting needs in health care systems, an increasingly common issue with the increase in hospital-based health systems.^5,6^ The findings should push the VA to consider outside access and use for new enrollees.

## Notes

Supplement 1. **eAppendix.** Veterans Affairs Survey of Enrollees

Supplement 2.
Data Sharing Statement
