# Supplementary material for: Association Between Self-Reported Health and Reliance on Veterans Affairs for Health Care Among Veterans Affairs Enrollees
Source: JAMA Netw Open. 2023 Jul 17;6(7):e2323884. doi: 10.1001/jamanetworkopen.2023.23884 (PMC10352854; doi:10.1001/jamanetworkopen.2023.23884)
Supplement: Supplement 1. — eAppendix. Veterans Affairs Survey of Enrollees [file jamanetwopen-e2323884-s001.pdf]

Supplemental Online Content

Rose L, Schmidt A, Gehlert E, Graham LA, Aouad M, Wagner TH. Association between self-reported health and reliance on Veterans Affairs for health care among Veterans Affairs enrollees. *JAMA Netw Open*. 2023;6(7):e2323884. doi:10.1001/jamanetworkopen.2023.23884

eAppendix. Veterans Affairs Survey of Enrollees

This supplemental material has been provided by the authors to give readers additional information about their work.

© 2023 Rose L et al. *JAMA Network Open.*

eAppendix. Veterans Affairs Survey of Enrollees

The VA Survey of Enrollees is an annual survey conducted by a third-party agency. An extensive summary of results from the survey is posted publicly (e.g. https://www.va.gov/VHASTRATEGY/SOE2021/2021_Enrollee_Data_Findings_Report-508_Compliant.pdf). The survey data includes information on the health status, insurance, utilization of VA and community health care, attitudes and perceptions towards VHA services, socioeconomic demographics, as well as other data that is not available in any other VHA database. The survey also inquires about enrollees' recent utilization of non-VA health care services paid by VA. These survey data play a crucial role in shaping policy decisions regarding Veteran health care and serve as a tool for VHA to make projections on enrollment, utilization, and cost.

The survey company uses VHA enrollment data to form the initial sample. The sample is stratified based on geographical health care markets, enrollee type, and priority groups, and for the purposes of analysis, Priority Groups 1-3 are classified into one stratum, Priority Groups 4-6 into a second stratum, and Priority Groups 7-8 into a third stratum. Individuals with missing data in these fields are dropped, along with individuals missing sex or having extreme values of age.

The survey is weighted to be representative of the VA population, including accounting for non-response bias. Surveys are conducted by mail and online, with a response rate around 30 percent.

In this analysis, we focused on the survey items on health insurance, reliance on VA for health care, and self-reported health. The exact question wording and possible response categories for these questions are as follows:

- "Are you covered by Medicare?" (Yes/No)
- "Do you receive your Medicare coverage through a Medicare Advantage plan?" (Yes/No)
- "Are you currently covered by Medicaid for any of your health care?" (Yes/No)
- "Are you currently covered by TRICARE?" (Yes/No)
- "Are you currently covered by any other individual or group health plan?" (Yes/No)
- "Please complete the following statement. I use VA services to meet…" ("all of my health care needs", "most of my health care needs", "some of my health care needs", "none of my health care needs")
- "Compared with other people your age, would you say your health is…" (Poor/Fair/Good/Very Good/Excellent)
    - In 2019 only, this was worded as: "In general, how would you rate your physical health?" (Poor/Fair/Good/Very Good/Excellent)

© 2023 Rose L et al. *JAMA Network Open.*
