# Supplementary material for: Association Between Self-Reported Health and Reliance on Veterans Affairs for Health Care Among Veterans Affairs Enrollees
Source: JAMA Netw Open. 2023 Jul 17;6(7):e2323884. doi: 10.1001/jamanetworkopen.2023.23884 (PMC10352854; doi:10.1001/jamanetworkopen.2023.23884)
Supplement: Supplement 2. — Data Sharing Statement [file jamanetwopen-e2323884-s002.pdf]

Data Sharing Statement

Rose. Association Between Self-Reported Health and Reliance on Veterans Affairs for Health Care Among Veterans Affairs Enrollees. *JAMA Netw Open*. Published online July 17, 2023.
doi:10.1001/jamanetworkopen.2023.23884

Data
Data available: No
